# Environmental impact of Norwegian self-selected diets: comparing current intake with national dietary guidelines and EAT-Lancet targets

**DOI:** 10.1017/S1368980024000715

**Published:** 2024-03-25

**Authors:** Julie Marie Lengle, Marie Michaelsen Bjøntegaard, Monica Hauger Carlsen, Sepideh Jafarzadeh, Lene Frost Andersen

**Affiliations:** 1 Department of Nutrition, University of Oslo, Oslo 0316, Norway; 2 SINTEF Ocean, Trondheim, Norway

**Keywords:** Environmental impact, Food-based dietary guidelines, Impact reduction, LCA, Sustainable diet, Scenario analysis

## Abstract

**Objectives:**

Dietary environmental impact in a Norwegian adult population was estimated for six environmental impact categories. Moreover, environmental benefits of scenario diets complying with the Norwegian Food-Based Dietary Guidelines (FBDG) and the EAT-Lancet reference diet were assessed.

**Design:**

The current diet of Norwegian adults was estimated according to 24-h dietary recall data from a national dietary surveillance survey (Norkost 3). Scenario diets were modelled to represent the Norwegian FBDG and the EAT-Lancet healthy reference diet. Dietary environmental impact in terms of global warming potential, freshwater and marine eutrophication, terrestrial acidification, water use and transformation and use of land was estimated for the current and scenario diets using environmental impact data representative of the Norwegian market. Significant associations between impact and gender/educational attainment were assessed at *P* < 0·05.

**Setting:**

Norway.

**Participants:**

Adults (*n*=1787) aged 18–70 years who participated in the Norkost 3 survey (2010–2011).

**Results:**

Environmental impact varied significantly by gender and educational attainment. The food groups contributing most to environmental impact of Norwegian diets were meat, dairy, beverages, grains and composite dishes. Compared with the current Norwegian diet, the FBDG scenario reduced impacts from 2 % (freshwater eutrophication) to 32 % (water use), while the EAT-Lancet scenario reduced impacts from 7 % (marine eutrophication) to 61 % (land use). The EAT-Lancet scenario resulted in 3–48 % larger reductions in impact than the FBDG scenario.

**Conclusions:**

The Norwegian FBDG, while not as environmentally friendly as the EAT-Lancet reference diet, can still be an important tool in lessening environmental burden of Norwegian diets.

The global food system is a major driver of climate change, land-use change and biodiversity loss, depletion of freshwater resources and pollution of aquatic and terrestrial ecosystems through nitrogen and phosphorus run-off from fertilizer and manure application^(1)^. Commitments made by the global community, including the Paris Climate Agreement and the United Nations Sustainable Development Goals, aim to guide the transition towards sustainable development^(2,3)^. The EAT-Lancet Commission Report on Healthy Diets from Sustainable Food Systems provides targets for diets that support health and food production systems that support environmental sustainability. These global production systems targets can be scaled down to an individual or national level using an equal per capita approach, and in this way serve as helpful tools to measure progress^(4,5)^. For example, despite the Nordic region’s strong commitment to the United Nations Sustainable Development Goals and other global goals, the impacts of Nordic food systems still far exceed four out of five food systems targets (greenhouse gas emissions, cropland use, nutrient application and biodiversity loss)^(5,6)^. Awareness of these transgressions allows for the discovery of potential mitigation pathways.

Previous research has pointed to environmental benefits of shifts to diets following national food-based dietary guidelines (FBDG), global dietary guidelines and/or healthy diet patterns, such as the aforementioned EAT-Lancet healthy reference diet^(5,7–10)^. Assessing the sustainability of current diets is necessary in order to determine the viability of proposed mitigation pathways, such as those to alleviate the environmental burden of diets in the Nordic region^(5)^. Earlier studies have estimated the environmental impact of Norwegian diets based on food balance sheets and using global databases of environmental data^(5,7,8)^. The main objective of this study was therefore to estimate the dietary environmental impact of Norwegian adults across several impact categories (global warming potential, freshwater and marine eutrophication, terrestrial acidification, water use and land use), using individual consumption data from a national dietary survey and data from a nationally developed environmental database. Moreover, analyses of environmental impacts across genders and levels of educational attainment were conducted. Further, the potential environmental benefits of a transition from the current diet towards diets following the Norwegian FBDG and the EAT-Lancet healthy reference diet were assessed.

## Materials and methods

### Current Norwegian diet

Dietary intake information for the current Norwegian diet was derived from Norkost 3, a national dietary survey conducted in 2010–2011^(11)^. A representative sample of 5000 individuals aged 18–70 years, born in Norway, Sweden or Denmark, was drawn from the Norwegian Population Register; 37 % agreed to participate. Two randomly distributed 24-h dietary recalls were conducted over telephone, with at least 4 weeks separation. Interviewers entered all responses directly into the nutrition calculation software system (KBS). The survey is described in more detail elsewhere^(12)^. Daily means over two consumption days were calculated for each participant and food intake was estimated per person (per day and per 10 MJ). Dietary supplements were excluded from the analysis. An overview of average daily consumption among survey participants is included in Table 1.

**Table 1:** Description of the current Norwegian diet (Norkost 3, 18–70 years), the Norwegian Food-Based Dietary Guidelines (FBDG)^(4,13)^ and the EAT-Lancet healthy reference diet (EAT-Lancet)

Food group	Current diet*	Norwegian FBDG†	EAT-Lancet	Possible range, g/d†
Grains, grain products	218 g (whole grains: 64 g)	≥4 portions whole grain products (women: ≥ 70 g, men: ≥ 90 g whole grains)/d.	232 g	0–60 % of total energy content††
Potatoes	68 g	Belong in a healthy diet.	50 g	0–100 g
Vegetables	150 g	≥2–3 portions (∼250 g)/d.	300 g	200–600 g
Fruit	271 g (incl. 100 g juice)	≥2–3 portions (∼250 g)/d. Max 100 g can be juice.	200 g	100–300 g
Legumes‡	12 g	Belong in a healthy diet.	75 g	0–150 g
All nuts	3 g	One small handful (20 g) unsalted nuts/d.	50 g	25–75 g‡‡
Red meat§	117 g	≤700–750 g/week. Choose lean products.	14 g	0–28 g
White meat||	34 g	Preferable to red meat. Choose lean products.	29 g	0–58 g
Eggs	25 g	No advice.	13 g	0–25 g
Fish, shellfish¶	73 g	365–545 g fish/week. ≥ 245 g should be oily fish.	28 g	0–100 g
Milk products	373 g	3 portions lean dairy/d.	250 g	0–500 g§§
Cooking fats	26 g	Choose oils, liquid margarine and soft margarine over hard margarine and butter.	52 g	20–92 g||||
Snacks, sweets	60 g (added sugars: 40 g)**	Not recommended.	31 g	0–31 g¶¶
Beverages	1085 g	Choose water when thirsty. Sugar-sweetened beverages and alcohol not recommended. Positive health effects of coffee and tea at reasonable consumption levels.	–	

* Mean daily intake in the Norkost 3 survey^(11)^. Mean energy consumption in the survey was 9·3 MJ (median 8·7 MJ), excluding dietary supplements.

† This table describes recommended content in the official Norwegian FBDG and the EAT-Lancet healthy reference diet. Content of the modelled scenarios is described in the Supplement, Tables S1–S2.

‡ Dry weight. Including pulses and soya products. Excluding peanuts (included under ‘All nuts’).

§ Raw weight, bone-free. The recommendation provided by the Norwegian FBDG is more commonly expressed in cooked weight (≤ 500 g/week).

|| Raw weight, bone-free.

¶ Raw weight, bone-free. The recommendation provided by the Norwegian FBDG does not specify if this amount is raw or cooked weight. Cooked weight was assumed and converted to raw weight using a conversion factor of 1·213 (the average of the conversion factors for fatty and lean fish provided in the report ‘Measurements, weight and portion sizes for food products’^(14)^).

** Including savory snacks, sweets and cakes.

†† Total grains (all whole grains), raw weight.

‡‡ Including both tree nuts and peanuts. Possible range includes a minimum of 24 g of tree nuts.

§§ Whole milk equivalents.

|||| Including unsaturated oils, palm oil and lard. Palm oil and lard were excluded in the final modelled diet used in this analysis, due to infrequence of use in Norway.

¶¶ All sweeteners. Diet does not include other discretionary foods.

### Diet scenario based on the Norwegian Food-Based Dietary Guidelines

The aim of this scenario was to model a singular plausible diet that follows the Norwegian FBDG, published in 2014 by the Norwegian Directorate of Health^(14)^. See Table 1 for a summary of the recommendations and online supplementary material, Supplemental Table S1 for detailed information on content of the diet scenario. 10 MJ was chosen as this is the approximate daily reference energy requirement of an average adult with a moderate physical activity level across all ages and genders^(15)^. For food groups with quantitative recommendations (grains, fruits, vegetables, nuts, fish and dairy), intake was set to follow these. For food groups with semi-quantitative (i.e. upper limit only) (red meat) or qualitative recommendations (white meat, fats, beverages), intake was based on average consumption observed in Norkost 3^(11)^ and on macronutrient recommendations^(15)^. For food groups with vague advice or no advice (potatoes, legumes and eggs), recommendations from other Nordic countries were considered along with observed intake^(16,17)^. Individual foods chosen in the scenario diet were selected based on observed intake in Norkost 3. To create a more realistic diet, discretionary foods and beverages were added in the amount suggested by the Danish FBDG (∼5 E%)^(16)^; this is about a third of the amount consumed in Norkost 3. Coffee and tea were also included in the diet in amounts similar to observed intake and drinking water in the amount recommended by the Nordic Nutrition Recommendations 2012^(15)^.

### Diet scenario based on the EAT-Lancet healthy reference diet

The modelled diet scenario for the EAT-Lancet healthy reference diet was formed based on the report ‘Food in the Anthropocene: the EAT-Lancet Commission on healthy diets from sustainable food systems’^(4)^. See Table 1 for an overview of the reference diet and online supplementary material, Supplemental Table S2 for information on composition of the diet scenario. In this report, a 10·5 MJ diet is proposed with a target value and range of possible values for each food group in both grams and kJ. The reference diet represents a global average and should in practice be adapted to local food culture^(4)^. However, for the sake of this comparison, the reference diet target values were used and amounts were scaled to give a total energy intake of 10 MJ; the proposed diet is as such only one representation of a diet that fits within the suggested ranges. Recommended intake in grams for each food category was matched with a representative food to meet the suggested kJ goals. Foods selected for inclusion were similar to those used in the FBDG scenario and were as such commonly consumed foods in Norway. Lard and palm oil were excluded from the diet scenario, as these foods are uncommon in Norway. Unsaturated oils are 25 % each of olive, soybean, rapeseed and sunflower oil; peanut oil was excluded due to lack of data availability. Recommended grain amount was converted from whole grains to a combination of whole grains and grain products. Drinking water was included in the amount recommended by the Nordic Nutrition Recommendations 2012^(15)^; however, no other beverages or discretionary calories beyond those detailed in the reference diet were included in the diet scenario.

### Environmental impact database

As part of the NOR-Eden project at the University of Oslo, a database was compiled from published life cycle assessment studies.^1^ Values were included for six impact categories (IC): the global warming potential of greenhouse gases on a 100-year timescale (kg CO_2_-eq); acidification of soils (kg SO_2_-eq); eutrophication of freshwater (kg P-eq) and marine waters (kg N-eq); water use, specifically the consumption of *extracted water* ^(18)^(m^3^) and transformation and use of land (m^2^a). The majority of included environmental data were compiled from studies using the assessment method ReCiPe 2016^(18)^. Literature searches for LCA studies on food items representing the Norwegian habitual diet and food market (i.e. considering both type of food and geographical region of origin) were conducted in the period 2019–2022. Reference lists of included articles were screened for relevant material. Literature published before 2010 was excluded. All relevant literature was quality assessed based on the methodological procedures by Weidema and Wesnæs^(19)^, Agri-Footprint^(20)^ and EcoInvent^(21)^. The database included system boundaries from farm-to-fork, thereby including primary production, processing and packaging, international (if relevant) and domestic distribution/transportation, energy use for storage in wholesale, retail and in the home of the consumer and consumer preparation. Transportation from retail to household was excluded.

For many food items, the identified LCA literature had different system boundaries than those chosen in the present project (e.g. many LCA studies only assessed impacts up to farm gate, while the present project includes the system boundaries farm-to-fork). Thus, surrogate data were compiled from similar foods or from available databases^(21–23)^. If no relevant source data was found, inventory data from the publications identified in the literature search were used to model the missing data in SimaPro (version 9.0.0.49)^(24)^, with processes from Agri-footprint^(20,23)^ or EcoInvent^(21)^. If the original sourced LCA data did not include waste, these data gaps were not filled due to missing data at time of compilation. These data gaps most likely apply mainly to the retail and household lifecycle stages.

The IC values were incorporated into the food composition and nutrition calculation system KBS at the Department of Nutrition at the University of Oslo. Environmental impacts are expressed as IC values per 100 g edible food item (i.e. excluding peel, bones). IC values for composite dishes were calculated based on recipes in KBS. Values were automatically adjusted for weight changes during cooking. Environmental and nutritional impacts of heat treatment were added when relevant.

### Estimation of environmental impact of current and scenario diets and statistics

The analysis was performed in two steps. In the first step, environmental impacts of the current diet were estimated on an individual level for all participants of the dietary survey, based on foods as reported consumed (farm-to-fork); i.e. including composite dishes, heat treatment and cooking-related weight changes. Impact of the current diet was estimated in ‘cooked’ form to capture as much of the life cycle as possible. In the second step, when comparing diet scenarios, environmental impacts of the current diet were estimated for the ‘raw’ form of the included foods; this facilitated comparison of the diet scenarios as the EAT-Lancet reference diet is provided in raw weight. Environmental impacts of the scenario diets were thus estimated based on foods in raw or ‘unprepared’ form (farm-to-retail).

Individual intake of each food was multiplied by its impact value per 100 g and impact contributions from all consumed foods were summed to give total dietary environmental impact. The difference in environmental impact for the current diet was < 1 % when calculated with farm-to-fork system boundaries, compared with farm-to-retail system boundaries. Results are presented as means, medians and quartiles in kg CO_2_-eq, g P-eq, g N-eq, g SO_2_-eq, m^3^ and m^2^a. Results for global warming potential, water use and land use were compared to the EAT-Lancet environmental boundaries (converted from global to per-capita boundaries by Wood et al.^(5)^).

Spearman rank correlation coefficients were calculated to examine the relationship between IC. Two-sample Wilcoxon rank test and Kruskal-Wallis equality-of-populations rank test were used to test for differences in IC values across genders and educational attainment levels, respectively. Multivariate linear regressions were used to test for associations between IC values, gender and educational attainment, after adjustment for energy consumption in MJ/d (see online supplementary material, Supplemental Table S5). Significance level was set to 0·05. Calculations and statistical tests were performed in STATA MP 17. Figures were created using the ggplot2 package in R version 4.2.2.

## Results

### Characteristics of the sample

Dietary environmental impacts from farm-to-fork were assessed for a sample of 1787 Norwegian adults. Overall, the sample was split evenly between males and females, with a slight majority of females (52 %). The majority of respondents were between the ages of 30 and 59 years (63 %), possessed a university degree (53 %) and had a BMI within the normal range (mean 25·4 kg/m^2^).

### The environmental impact of the current Norwegian diet

Daily environmental impact of the current diet is presented in Table 2. Significant (*P* < 0·0001) correlations were found between values for global warming potential and freshwater and marine eutrophication, terrestrial acidification and land use, ranging from 0·76 to 0·92 (see online supplementary material, Supplemental Table S3). Water use had the lowest correlation with global warming potential (0·52; *P* < 0·0001). Online supplementary material, Supplemental Table S4 compares impacts associated with the current Norwegian diet to environmental boundaries based on the EAT-Lancet targets^(5)^. Environmental impact of Norwegian consumption was estimated to be 2·5 times the carbon footprint boundary and just above the land use boundary, but below the water use boundary.

**Table 2:** Daily environmental impact of current dietary consumption in a sample of 1787 Norwegian adults aged 18–70 years, stratified by gender and educational attainment. System boundaries are farm-to-fork; incl. Cooking, excl. Avoidable waste at the retail and household levels

	All			Men			Women			Low educational attainment†			Moderate educational attainment†			High educational attainment†		
	*n* 1787 9·3 (8·7) MJ‡			*n* 862 10·7 (10·4) MJ‡			*n* 938 7·9 (7·7) MJ‡			*n* 287 9·1 (8·6) MJ‡			*n* 559 9·3 (8·5) MJ‡			*n* 938 9·3 (8·9) MJ‡		
	Mean	Median	Q1;Q3	Mean	Median	Q1;Q3	Mean	Median	Q1;Q3	Mean	Median	Q1;Q3	Mean	Median	Q1;Q3	Mean	Median	Q1;Q3
GWP kg CO_2_-eq	4·7	4·2	3·3; 5·6	5·5*	5·0	3·9; 6·5	4·0*	3·7	2·9; 4·7	4·6	4·1	3·1; 5·7	4·8	4·2	3·2; 5·8	4·7	4·3	3·4; 5·6
FE g P-eq	1·1	1·0	0·8; 1·3	1·2*	1·1	0·9; 1·5	0·9*	0·9	0·7; 1·1	1·0*	1·0	0·7; 1·3	1·1*	1·0	0·7; 1·2	1·1*	1·0	0·8; 1·3
ME g N-eq	4·5	4·2	3·2; 5·3	5·2*	4·8	3·8; 6·3	3·8*	3·6	2·8; 4·5	4·4	4·1	3·0; 5·3	4·5	4·0	3·1; 5·3	4·5	4·3	3·3; 5·4
TA g SO_2_-eq	49	42	30; 60	57*	50	37; 70	41*	36	27; 49	48	41	29; 62	50	41	30; 62	48	43	31; 57
WU m^3^	0·5	0·5	0·3; 0·7	0·6*	0·6	0·4; 0·8	0·5*	0·4	0·3; 0·6	0·5*	0·5	0·3; 0·6	0·5*	0·5	0·3; 0·7	0·5*	0·5	0·4; 0·7
LU m^2^a	5·3	4·3	3·2; 6·2	6·3*	5·3	3·9; 7·5	4·3*	3·7	2·8; 4·9	5·3	4·5	3·0; 6·3	5·6	4·3	3·2; 6·4	5·1	4·3	3·3; 6·0

Q1, first quartile; Q3, third quartile; GWP, global warming potential; CO_2_-eq, carbon dioxide equivalents; FE, freshwater eutrophication; P-eq, phosphorous equivalents; ME, marine eutrophication; N-eq, nitrogen equivalents; TA, terrestrial acidification; SO_2_-eq, sulfur dioxide equivalents; WU, water use; m3, cubic meters; LU, land use; m^2^a, square meters of land per year.

* Two-sample Wilcoxon rank test was used for gender and Kruskal–Wallis equality-of-populations rank test was used for level of educational attainment. Results marked with * indicate a significant difference (*P* < 0·05) between groups (men/women and low/moderate/high educational attainment). Linear regression analyses were also performed to examine the energy-adjusted relationship between impact category values and subgroups gender and level of educational attainment. Results are presented in online supplementary material, Supplemental Table S5.

† Educational attainment: Low (primary school or lower), moderate (high school, trade school) and high (university and above).

‡ Average (median) daily energy intake.

### Contribution of food groups to environmental impact of current Norwegian diet

**Fig. 1:**
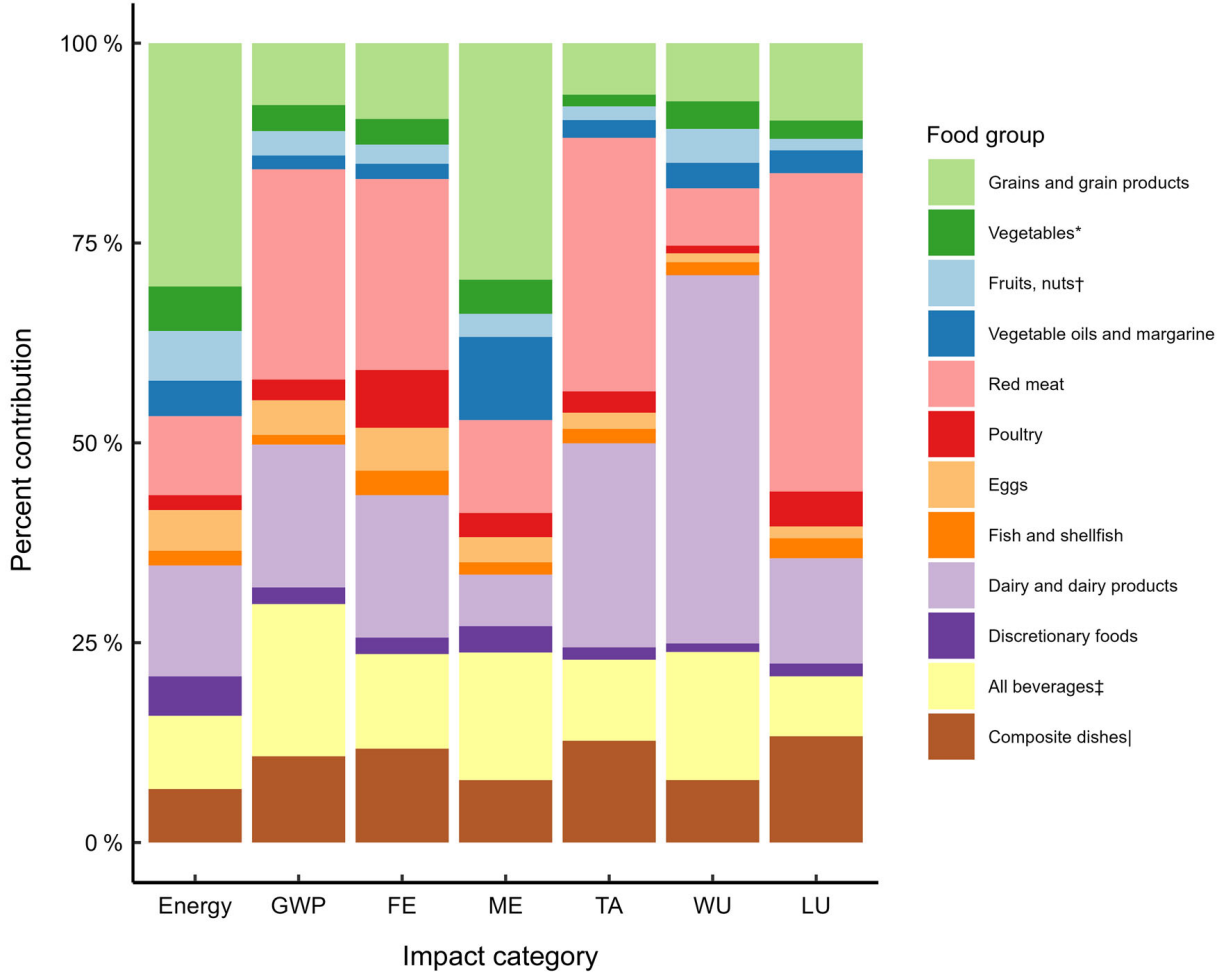
Relative contributions (% of total/day) of food categories to daily energy consumption and global warming potential (kg CO_2_-eq), freshwater eutrophication (g P-eq), marine eutrophication (g N-eq), terrestrial acidification (g SO_2_-eq), water use (m^3^) and land use (m^2^a) in a sample of Norwegian adults aged 18–70 years. System boundaries are farm-to-fork; incl. cooking, excluding avoidable waste at the household level. *All vegetables including potatoes, legumes (excluding peanuts). †Fruits, nuts, berries and seeds including peanuts. ‡All beverages including juice, coffee, tea, alcohol, soft drinks. |Composite dishes including pizza, lasagna, burgers, wraps, etc. GWP, global warming potential (kg CO_2_-eq); FE, freshwater eutrophication (g P-eq); ME, marine eutrophication (g N-eq); TA, terrestrial acidification (g SO_2_-eq); WU, water use (m3); LU, land use (m^2^a).

The food groups contributing most to the environmental impact of Norwegian diets were meat, dairy, beverages, grains and composite dishes (Fig. 1). Meat was the single food group contributing most to the global warming potential (29 %), freshwater eutrophication (31 %), terrestrial acidification (34 %) and land use (44 %). Red meat accounted for 77–91 % of meat’s impacts for each IC. Dairy consumption accounted for nearly half of total water use and 7–25 % of the other IC totals. Beverages stood for 15–20 % of total global warming potential, marine eutrophication and water use. Grains were the food group contributing most to marine eutrophication (29 %), while composite dishes contributed most to land use and terrestrial acidification (13 %).

Other animal products, such as fish and eggs, contributed marginally to dietary impacts. On the whole, contribution of plant-sourced foods to overall dietary impact remained relatively low compared to animal-sourced foods; an exception is seen for marine eutrophication, where grains, vegetables, fruits and vegetable oils stood for 47 % of the total impact, compared with 12–18 % of total impact for other IC.

### Variation in environmental impact of current diet across population subgroups

Dietary environmental impact varied according to gender and educational attainment (Table 2 and see online supplementary material, Supplemental Table S5). Men had significantly higher dietary environmental impact than women across all IC, while individuals with low or moderate educational attainment had significantly lower dietary freshwater eutrophication and water use than individuals with high educational attainment. After energy adjustment, a significant association was found between male gender and increased global warming potential, freshwater eutrophication, terrestrial acidification and land use. Among these IC, the smallest difference in energy-adjusted mean between genders was seen for global warming potential (1 %), while the largest mean difference was seen for land use (7 %). After energy adjustment, both low and moderate educational attainment had a significant association with decreased freshwater eutrophication, compared with high educational attainment. Further, low educational attainment had a significant association with decreased water use and moderate educational attainment with decreased marine eutrophication, compared with high educational attainment. However, the difference in energy-adjusted means was small (up to 1 %).

### Environmental impact of current diet compared with healthy and sustainable diet scenarios

**Fig. 2:**
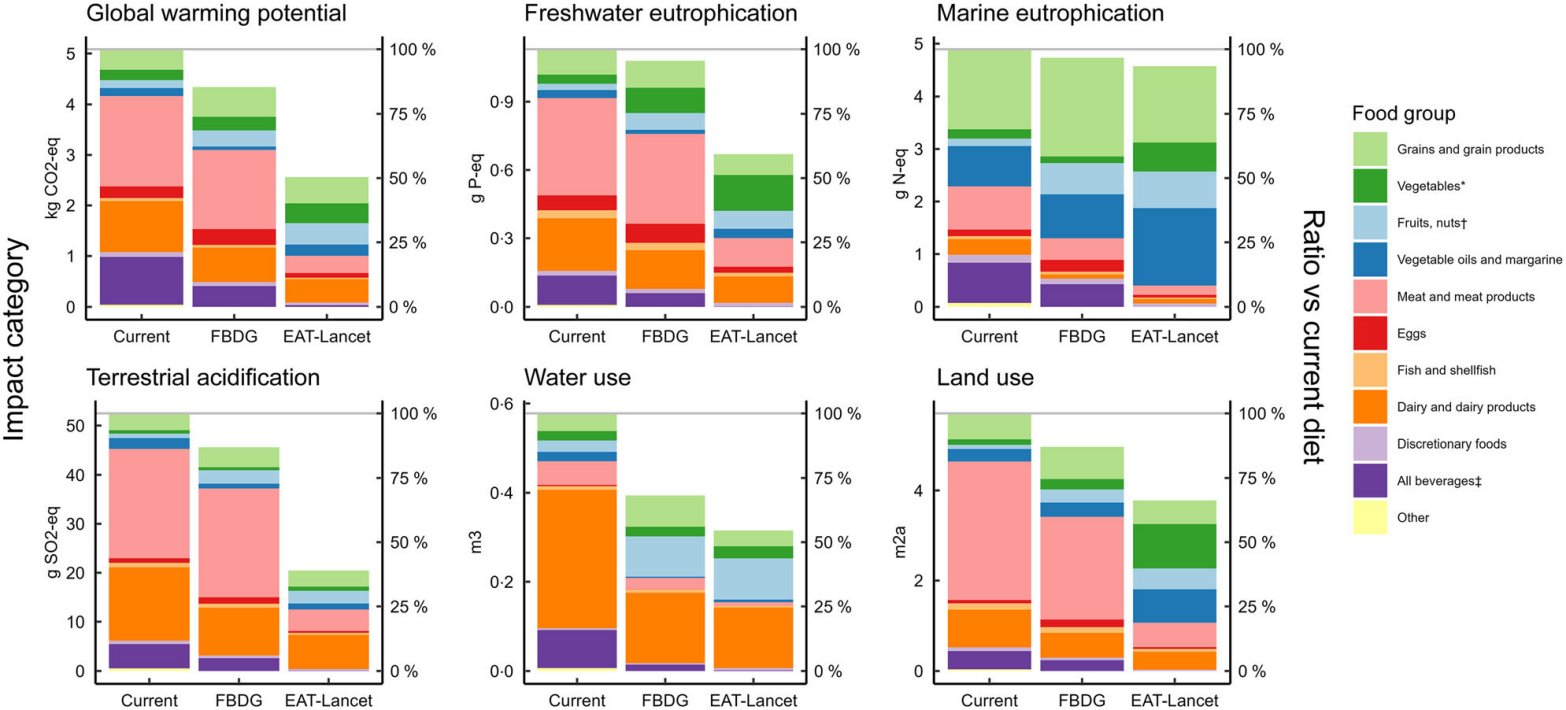
Relative contributions (% of total/day) of food categories to daily diet-associated global warming potential (kg CO_2_-eq), freshwater eutrophication (g P-eq), marine eutrophication (g N-eq), terrestrial acidification (g SO_2_-eq), water use (m^3^) and land use (m^2^a) in modelled diets representing the Norwegian Food-Based Dietary Guidelines (FBDG) and the EAT-Lancet healthy reference diet (EAT-Lancet), compared with that of the current diets of a sample of Norwegian adults aged 18–70 years. *All vegetables including potatoes, legumes (excluding peanuts). †Fruits, nuts, berries and seeds including peanuts. ‡All beverages including juice, coffee, tea, alcohol and soft drinks

Tables 3 and 4 describe food intake and environmental impact (from farm-to-retail) per 10 MJ in the three diet scenarios, and Fig. 2 compares the environmental impacts of the diet scenarios. For five out of six IC a gradient can be seen, with highest overall impact from the current diet to lowest overall impact from the EAT-Lancet scenario diet. Across IC, the FBDG scenario had 2–32 % lower impact than the current diet, while the EAT-Lancet scenario diet had 7–61 % lower impact. Marine eutrophication was the IC for which the EAT-Lancet scenario diet was least effective in reducing compared with the current diet (7 % reduction compared with 34–61 % for other IC), while the FBDG scenario was least effective in reducing both marine and freshwater eutrophication, due to high content of both plant-based foods and dairy (3 % and 2 % reductions, respectively, compared with 13–32 % for other IC). The FBDG scenario most effectively reduced water use (32 % reduction) and the EAT-Lancet scenario terrestrial acidification (61 % reduction). Overall, the EAT-Lancet scenario resulted in 3–48 % larger reductions in impact than the FBDG scenario.

**Table 3:** Intake in g/10 MJ of food groups in the diet of a sample of Norwegian adults aged 18–70 years and in modelled diet scenarios representing the Norwegian Food-Based Dietary Guidelines (FBDG) and the EAT-Lancet healthy reference diet (EAT-Lancet). See online supplementary material, Supplemental Fig. S1 for a graphical comparison of % energy contribution per food group in the diet scenarios

Food group	Current diet*	Norwegian FBDG	EAT-Lancet
	g/10 MJ†	g/10 MJ†	g/10 MJ†
Grains and grain products	234	356	274
Potatoes	73	100	48
Vegetables	161	300	286
Legumes‡	11	10	72
Fruits and berries	184	300	191
Tree nuts and seeds	5	20	48
Dairy and dairy products, all	408	350	160§
Milk and other dairy	362	330	140
Cheese	46	20	20
Meat and meat products, total	162	120	42
Red meat (beef, lamb, pork, etc.)	126	100	14
Poultry	36	20	28
Eggs	26	24	12
Fish and shellfish	78	70	27
Vegetable oils and margarine	21	26	44
Discretionary foods, all	64	22	25
Beverages, all	2496	1730	1100
Water	1223	1000	1000
Juice	188	0	0
Sweetened beverages	179	50	100
Alcoholic beverages	152	0	0
Coffee and tea	754	680	0
Spices, sauces, other	26	0	0

* Mean daily intake in the Norkost 3 survey^(11)^ adjusted to 10 MJ. Mean energy consumption in the survey was 9·3 MJ (median 8·7 MJ), excluding dietary supplements.

† Food amounts are primarily expressed in raw weight, except for prepared foods such as bread, sausages, cold cuts, smoked or canned fish products and beverages.

‡ All legumes excluding peanuts. Peanuts are included under tree nuts and seeds.

§ Converted from whole milk equivalents using milk equivalent factors 1·0 (yoghurt) and 5·0 (cheese). From Wood A, Gordon LJ, Röös E et al. (2019) Erratum: Nordic food systems for improved health and sustainability – baseline assessment to inform transformation. Stockholm Resilience Centre. https://www.stockholmresilience.org/download/18.8620dc61698d96b1901719c/1561013818461/Erratum_Nordic%20report_14-6-19.pdf. Accessed 29 March 2023.

**Table 4:** Daily environmental impact of average dietary consumption per 10 MJ in a sample of Norwegian adults aged 18–70 years and in modelled diet scenarios representing the Norwegian Food-Based Dietary Guidelines (FBDG) and the EAT-Lancet healthy reference diet (EAT-Lancet). System boundaries are farm-to-retail

	Current diet *n* 1787	Norwegian FBDG	EAT-Lancet
	Mean	Mean	Mean
GWP kg CO_2_-eq	5·08	4·33	2·56
FE g P-eq	1·13	1·08	0·67
ME g N-eq	4·89	4·73	4·56
TA g SO_2_-eq	52·5	45·6	20·4
WU m^3^	0·58	0·39	0·31
LU m^2^a	5·70	4·96	3·77

GWP, global warming potential; CO_2_-eq, carbon dioxide equivalents; FE, freshwater eutrophication; P-eq, phosphorous equivalents; ME, marine eutrophication; N-eq, nitrogen equivalents; TA, terrestrial acidification; SO_2_-eq, sulphur dioxide equivalents; WU, water use; m3, cubic meters; LU, land use; m^2^a, area time.

In both the current diet and FBDG scenario, meat and dairy products contributed most to environmental impact. However, grains, fruits and nuts contributed more noticeably to marine eutrophication and water use in the FBDG scenario than in the current diet. For the EAT-Lancet scenario, dairy and grains contributed most to impacts across IC. Fruits, nuts, vegetables (notably legumes), meat and vegetable oils also contributed markedly.

## Discussion

This is the first study to estimate the environmental impact of self-selected dietary intake among adult Norwegians across several IC and population subgroups. Daily environmental impact varied significantly between genders and levels of educational attainment. Intake of meat, dairy, beverages, grains and composite dishes mainly determined dietary environmental impact. A modelled diet complying with the Norwegian FBDG led to a 2–32 % reduction (e.g. depending on IC) in environmental impact compared with the current diet. Further, a modelled diet following the EAT-Lancet healthy reference diet guidelines led to a 7–61 % reduction compared with the current diet.

### Environmental impact of current Norwegian diets

Most published studies on the environmental impact of Nordic diets have been limited to carbon footprint^(25–27)^. Studies from Sweden and Denmark using self-reported dietary data from adults have found estimated daily per capita dietary carbon footprints between 5·0–5·5 kg CO_2_-eq^(28,29)^ and 4·2–5·3 kg CO_2_-eq^(9,10)^, respectively. These results are comparable with what was observed in the present study (4·7 kg CO_2_-eq). A few studies have previously estimated carbon footprint of Norwegian diets^(5,7,8)^. Behrens et al.^(8)^ estimated ∼3·8 kg CO_2_-eq/person/day, whereas Springmann et al.^(7)^ (4·8 kg CO_2_-eq) and Wood et al.^(5)^ (∼4·9 kg CO_2_-eq) found values more similar to what was observed in the present study. The differences in estimated carbon footprint observed across studies are likely due to common methodological disparities that affect estimates and confuse comparisons between studies, e.g. source of dietary and environmental data, choice of system boundaries, food waste adjustment and study population. However, there is a general consensus that the carbon footprint of Nordic diets is above the average for high-income country populations^(5,7,8)^.

Compared with environmental boundaries downscaled from the EAT-Lancet targets using an equal per capita approach^(5)^, the environmental impact of Norwegian consumption as estimated in the present study is 2·5 times the carbon footprint boundary and slightly above the land use boundary, but below the water use boundary. These conclusions are similar to those made by Wood et al. in their appraisal of the impact of Nordic consumption, though land use was estimated to be 50–100 % higher than found in the present study^(5)^. Two additional studies have more thoroughly estimated dietary environmental impact of Swedish adults in relation to planetary boundaries and reached similar conclusions to those found in the present study, though their absolute values for carbon footprint and land use were slightly higher than in the present study, and for water use notably lower^(6,30)^. There are several ways to downscale the global EAT-Lancet targets for comparison of individual-, regional- or national-level impacts. The equal per capita approach is based on the idea of sharing allowances of environmental impacts equally across every person of the global population, but other approaches could, for example, account for regional variation in production conditions, traditions and habits^(5)^. Comparison with equal per capita boundaries is therefore limited, but is useful to indicate the scale of change needed. As suggested by these results, the degree of change needed to reduce environmental footprint of Nordic consumption below the environmental boundaries is particularly large for carbon footprint.

### Food group contributions to environmental impact in the current diet

Meat, dairy, beverages, grains and composite dishes mainly determined dietary carbon footprint in the present study, and animal products were responsible for the largest share of impacts for all IC except marine eutrophication and water use. Overall, the patterns of food group contributions to total dietary impact seen for global warming potential, freshwater eutrophication, terrestrial acidification and land use were largely comparable. These findings are in line with the significant correlations found between IC in this study and in previous research^(31,32)^. However, food group contributions to marine eutrophication differed from those seen for other IC in the present study, the largest difference being that plant-based foods (especially grains and vegetable oils) stood for the majority of marine eutrophication. These results are interesting, as water use is typically thought to be the IC that is most determined by plant-based foods, since, e.g. fruits, nuts, vegetable oils, rice, cereals and pulses can be more dependent on irrigation than animal foods^(7,31,33)^. High intake of bread in Norwegian diets likely plays a role in this finding; however, the larger overall contribution from plant-based foods compared with other studies is presumably due to the impact of source data from an LCA study of Norwegian margarine products^(34)^. Further, although meat contributed noticeably less to water use than to other IC in the present study, the high contribution of dairy products (46 %) compensated for this and animal products remained the main contributor.

The patterns seen for food group contributions to total dietary carbon footprint in the present study are similar to those seen in other Northern and Western European countries^(9,28,31,35,36)^. Similar results were found in Sweden and Denmark, though both had higher relative contributions of dietary carbon footprint from animal-sourced products^(9,28)^. This difference lessens markedly if the food group composite dishes (used only in the present study) is split into its respective parts, implying that the carbon footprint of composite dishes is mainly due to their content of meat and dairy products. Both countries’ diets also saw higher contributions from fruits and vegetables and Swedish diets from fish and shellfish, due to higher intake compared with current Norwegian diets.

### Variation in environmental impact of current diet across population subgroups

In the present study, Norwegian men had significantly higher dietary environmental impacts for all IC than women. The differences persisted after energy adjustment for carbon footprint, freshwater eutrophication, terrestrial acidification and land use. Although difference in energy intake is stressed as the main factor explaining differences in carbon footprint between genders^(27,37)^, a number of other studies have also discovered differences that persist after energy-adjustment^(33,36,38,39)^. In regards to other IC, a German study found higher land use from male diets after energy adjustment, while water footprint was shown to be higher among women^(33)^. In the aforementioned study as in the present study, higher proportional intake of animal-sourced foods, especially red and processed meat, among men largely explains differences in dietary carbon footprint and land use density between genders^(11)^.

Small differences were found in environmental impact across levels of educational attainment both before and after energy adjustment. For water use and freshwater and marine eutrophication, there was a significant relationship between possession of a university degree or higher and slightly increased dietary impacts (up to 1 %). Educational gradients in dietary environmental impacts have been discovered in previous research^(36,39,40)^. It has been theorised that higher educational level, and thereby higher income, may enable more frequent consumption of meat, fish and cheese, i.e. more expensive foods with higher environmental impacts^(5,39)^. However, in the present study, global warming potential, terrestrial acidification and land use, typically associated with meat and animal products, were similar between educational groups, as was consumption of these foods^(11)^. Intake of some plant-based foods (e.g. grains, fruits and vegetables) and beverages (e.g. juice, wine and tea) was higher among individuals with higher educational attainment in the dietary survey; these foods are more closely linked with the IC water use and freshwater and marine eutrophication, for which small differences were seen between educational backgrounds in the present study.

### Impact reduction by transitioning to diets following Norwegian Food-Based Dietary Guidelines and the EAT-Lancet healthy reference diet

Compared with the current diet, a modelled diet complying with FBDG had a lower content of meat, dairy products and discretionary foods, in combination with an increased content of grains, fruits, nuts and vegetables. These modelled dietary changes resulted in reductions in all IC. The reduction in meat (especially red meat) is largely responsible for the decrease in marine eutrophication and land use; this is in line with previous research asserting that reducing meat consumption is effective in lowering the environmental impact of the diet^(1,4,27)^. Though meat reduction was an important driver of impact reduction for all categories, reduction in dairy products stood for the greatest reductions in terrestrial acidification and water use, while beverages stood for the greatest decrease in global warming potential and freshwater eutrophication. As observed intake of coffee and tea was upheld in the FBDG scenario, this reduction in environmental impact seen in relation to beverages is due to reduced intake of sweetened beverages, alcohol and juice. Previous research in Sweden and Denmark has also pointed to the potential environmental benefits of a shift from current diets to ones that follow Nordic or national dietary guidelines^(9,29,41,42)^.

In the present study, a modelled diet following the EAT-Lancet reference diet recommendations was shown to have up to 48 % greater reductions in impacts compared with a modelled diet following the Norwegian FBDG and up 61 % lower impacts than the current diet. The differences in environmental impact between the FBDG and EAT-Lancet scenarios were substantial for global warming potential, freshwater and marine eutrophication, freshwater eutrophication, terrestrial acidification, water use and land use. The EAT-Lancet reference diet contains around one-fourth of the amount of meat consumed in the current diet, one-third of the amount of fish and half of the amount of dairy. The Norwegian FBDG also encourage consumption of lean dairy products and leave room for consumption of reasonable amounts of red meat, coffee and tea, while the EAT-Lancet reference diet does not. The decision to include coffee, tea and discretionary calories in the FBDG scenario, while excluding addition of these foods in the EAT-Lancet scenario, likely affected results. Though discretionary foods contributed little to the overall impact of all three diets (< 3 %), beverages had a clear contribution to impacts in both the current (7–19 %) and FBDG scenario diet (4–10 %). In the current diet, 50–70 % of these impacts were linked to coffee and tea, while 70–90 % of impacts from beverages were linked to coffee and tea in the FBDG scenario. Inclusion of coffee and tea in the observed amount in the EAT-Lancet scenario would thus have reduced the differences in impact between the scenario diets. The difference between impacts of the two scenario diets was, however, already minimal for marine eutrophication (4 %); further, neither diet led to a substantial reduction in marine eutrophication compared with the current diet (3–7 % reduction). Like many healthy guidelines, the FBDG and EAT-Lancet reference diet recommend increased consumption of plant-based foods, including unsaturated plant oils. Previous evidence suggests that the relatively high contribution of plant-based foods to levels of marine eutrophication, compared with that seen for other environmental IC, necessitates more dramatic changes in dietary patterns in order to reach significant reductions; this indicates a need for concurrent improvements in production methods^(4,43)^.

When comparing these results with the downscaled EAT-Lancet environmental boundaries, the FBDG scenario diet was shown to remain above the environmental boundary for carbon footprint, but reduce land use to the boundary and reduce water use further. Interestingly, although the EAT-Lancet scenario reduced all three IC further than the FBDG scenario, carbon footprint remained above the environmental boundary. This comparison is limited, as both diets are only represented by one scenario; however, these results may point to the importance of within-food group variation in environmental impact. The EAT-Lancet reference diet provides target values for intake of food groups, but largely does not specify individual food types (except for oils, where specific recommendations are made). Environmental impacts of similar foods are varied; impact differs between foods within the same food group and also depends on factors such as place of origin, production efficiency and seasonality^(43)^. The environmental impact of the final diet is thus dependent not only on overall food group composition but also on which individual foods are chosen. This conclusion has earlier been proposed as a potential avenue for reduction of food-related environmental impacts – food swaps between similar products may be promoted when larger and more rapid dietary transitions may not be palatable or possible^(43)^.

### Strengths and limitations

A major strength of this paper is the quality and national representativeness of the dietary data^(11)^. Further, the environmental impact database used in this study was comprehensive. The database contained information on six environmental IC, allowing for evaluation of potential trade-offs between indicators, and region-specific data were prioritised when building the database to better represent the Norwegian context. Regularly consumed foods were also prioritised, such that the resulting database had a high coverage of the foods that, in sum, give a high coverage of the energy intake in the population. Moreover, the use of commonly consumed foods in construction of the two dietary scenarios provided a realistic representation of national food preferences and increased cultural acceptability of the diet scenarios.

The present study has some important limitations. The dietary data are approximately ten years old; but on average, the data are considered to represent the Norwegian current diet fairly well because dietary changes at the population level are generally slow^(44)^. However, trends in Norwegian food supply data indicate slightly increasing consumption of meat and cheese and decreasing consumption of fish and fruit^(44)^. Furthermore, misreporting is commonly seen in dietary surveys among adults^(45)^; earlier estimations indicate that 16 % of respondents under-reported energy intake in the Norkost 3 study^(11)^. Moreover, the FBDG and EAT-Lancet healthy reference diet were represented by only one scenario each, while diets following these guidelines might be achieved in multiple ways. Future research should consider constructing multiple or weekly scenarios for each set of guidelines in order to increase variation and representativeness of the diet scenarios. Another limitation is that avoidable food waste at the retail and household level was not accounted for in the impact estimates. In 2015, it was estimated that 355 000 tons of avoidable food waste were generated in Norway from the food industry, wholesale, retail and households; most (61 %) was attributed to households^(46)^. Exclusion of avoidable food losses in this study has most likely led to an underestimation of dietary environmental impact. Finally, although the most suitable references were used when building the database, there will always be uncertainty in the IC values per food item due to differences in the methods applied, year of data collection for primary production, standard factors used, etc. For some foods, less LCA data were available, necessitating extrapolation of values from other foods or production systems and introducing more uncertainty to the data.

### Conclusion

The present study showed that the environmental burden of current Norwegian diets is high, with a carbon footprint similar to that seen in other Nordic countries. Men were found to have higher energy-adjusted dietary impacts than women for most IC. Results from the scenario analysis indicate that a transition towards diets following the Norwegian FBDG has the potential to decrease the environmental impact of Norwegian diets by up to one third across a range of IC, while following the EAT-Lancet healthy reference diet could decrease environmental impact of Norwegian diets by up to two-thirds.

These results suggest that the national FBDG, while not as environmentally friendly as some proposed healthy diets, can still be an important tool in the transition towards more sustainable Norwegian diets. Policy measures that could incentivise a greater uptake of existing FBDG include educating people in preparation of nutritionally adequate meals centering plant-based foods, adopting public procurement standards that are in line with FBDG and making sure policies from other governmental departments and ministries are aligned with and do not contradict the recommendations of FBDG.

## Supporting information

Lengle et al. supplementary material Lengle et al. supplementary material

## Footnotes

1 Manuscript in preparation.

## Supplementary material

For supplementary material accompanying this paper visit https://doi.org/10.1017/S1368980024000715
