# Supplementary material for: Environmental impact of Norwegian self-selected diets: comparing current intake with national dietary guidelines and EAT-Lancet targets
Source: Public Health Nutr. 2024 Mar 25;27(1):e100. doi: 10.1017/S1368980024000715 (PMC11010176; doi:10.1017/S1368980024000715)
Supplement: Lengle et al. supplementary material [file S1368980024000715sup001.docx]

***Public Health Nutrition***

Supplementary information

Title: **Environmental impact of Norwegian self-selected diets: comparing current intake with national dietary guidelines and EAT-Lancet targets**

***Supplementary tables and figures***

**Table S1** - Overview of the Norwegian Food-Based Dietary Guidelines^(1)^ and foods included in the modelled scenario.

**Table S2** - Overview of the EAT-Lancet healthy reference diet^(2)^ (at 10 MJ) and foods included in the modelled diet scenario.

**Table S3** - Correlation matrix of spearman correlation coefficients for greenhouse gas emission and terrestrial acidification, freshwater eutrophication, marine eutrophication, land use and water use for current dietary intake in a sample of Norwegian adults aged 18-70 years (n=1787, 2010-2011)^(3)^.

**Table S4** – Daily environmental impact of dietary consumption in a sample of 1787 Norwegian adults aged 18-70 years, and in modelled diets representing the Norwegian Food-Based Dietary Guidelines and the EAT-Lancet healthy reference diet, compared to environmental boundaries downscaled from the EAT-Lancet targets using an equal per capita approach^(4)^.

**Table S5** - Energy-adjusted relationships between daily dietary environmental impact and gender/level of educational attainment in a sample of Norwegian adults aged 18-70 years (n=1787, 2010-2011)^(3)^, results from multiple linear regression analysis.

**Figure S1** - Relative contributions (% of total/day) of food categories to daily energy consumption in a sample of Norwegian adults aged 18-70 years^(3)^ and in modelled scenario diets representing the Norwegian Food-Based Dietary Guidelines and the EAT-Lancet healthy reference diet. System boundaries are farm-to-retail.

| **Table S1.** Overview of the Norwegian Food-Based Dietary Guidelines^(1)^ and foods included in the modelled scenario. | | | | | | |
| --- | --- | --- | --- | --- | --- | --- |
| General | Varied diet with plenty of vegetables, fruit and berries, wholegrain products and fish, and limited amounts of processed meat, red meat, salt and sugar.  Balance between energy consumption and expenditure.  Physical activity ≥ 30 mins/day. | | | | | |
| **Guidelines:** | | **Included in scenario:** | | | | |
|  |  | *g/day* | *kJ/day* |  | *g/day* | *kJ/day* |
| Total |  | 3425 | 10000 |  | 3442 | 10000 |
| Grains and grain products | ≥ 4 portions whole grain products (70-90g whole grains)/day. | 356  (whole grains: 270) | 4611 | Brown rice, dry | 38 | 586 |
|  |  |  |  | Wholegrain bread | 175 | 1702 |
|  |  |  |  | Wholegrain crispbread | 30 | 574 |
|  |  |  |  | Rolled oats, dry | 75 | 1167 |
|  |  |  |  | Wholegrain pasta, dry | 38 | 582 |
| Potatoes | Belong in a healthy diet. | 100 | 340 | Winter potatoes, raw | 100 | 340 |
| Vegetables | ≥ 250g vegetables/day. | 300 | 341 | Capsicum, raw | 12 | 12 |
|  |  |  |  | Onion, raw | 30 | 41 |
|  |  |  |  | Broccoli, raw | 100 | 119 |
|  |  |  |  | Iceberg lettuce/ruccola, raw | 10 | 6 |
|  |  |  |  | Tomato, raw | 43 | 32 |
|  |  |  |  | Carrot, raw | 70 | 105 |
|  |  |  |  | Canned tomatoes | 25 | 22 |
|  |  |  |  | Cucumber, raw | 10 | 4 |
| Fruits and berries | ≥ 250g fruit and berries/day. Max 100g of this can be juice. | 300 | 673 | Orange, raw | 130 | 203 |
|  |  |  |  | Apple, raw | 85 | 169 |
|  |  |  |  | Banana, raw | 85 | 301 |
| Legumes | Belong in a healthy diet. | 10 | 130 | Green/brown lentils, dry | 10 | 130 |
| Nuts | 20g unsalted nuts/day. | 20 | 495 | Cashews, raw | 10 | 246 |
|  |  |  |  | Almonds, skin on, raw | 10 | 249 |
| Red meat (beef, lamb, pork, etc) | ≤ 700-750 g red meat and processed red meat products/week^a^. Choose lean meat and lean meat products. | 100 | 500 | Beef, 2% fat, raw | 50 | 226 |
|  |  |  |  | Ham roast, raw | 50 | 274 |
| Poultry | Advisable to replace some red meat with white meat if intake of red meat is high, choose lean products. | 20 | 94 | Chicken filet, skinless, raw | 20 | 94 |
| Eggs | No advice. | 24 | 148 | Egg, raw | 24 | 148 |
| Fish and shellfish | 365-545 g fish/week^b^.  ≥ 245 g should be oily fish. | 70 | 479 | Salmon, farmed, raw | 40 | 375 |
|  |  |  |  | Cod, wild, raw | 30 | 104 |
| Milk products (incl cheese, excl butter) | 3 portions lean dairy products/day. To cover iodine needs at least two of these portions should be milk, kefir or yoghurt. | 350 | 943 | Milk, 0,5% fat | 200 | 309 |
|  |  |  |  | Yoghurt, plain, 3% fat | 130 | 376 |
|  |  |  |  | White cheese, low-fat, 15% fat | 10 | 109 |
|  |  |  |  | Brown cheese, low-fat, 16% fat | 10 | 149 |
| Animal fats (incl butter and lard) | Choose cooking oils, liquid margarine and soft margarine over hard margarine and butter. | 0 | 0 |  |  |  |
| Vegetable oils and margarine |  | 26 | 753 | Low-fat margarine | 7 | 105 |
|  |  |  |  | Liquid margarine | 7 | 208 |
|  |  |  |  | Rapeseed oil | 6 | 220 |
|  |  |  |  | Olive oil | 6 | 220 |
| Snacks & sweets | Not recommended. | 22 | 405 | Potato chips, salted | 5 | 105 |
|  |  |  |  | Gummy candy mix, no chocolate | 7 | 114 |
|  |  |  |  | Chocolate | 5 | 101 |
|  |  |  |  | White sugar | 5 | 85 |
| Water | Choose when thirsty. | 1000 | 0 | Tap water | 1000 | 0 |
| Sweetened beverages | Not recommended. | 50 | 90 | Coca cola, sugar-sweetened | 50 | 90 |
| Alcoholic beverages | Not recommended. | 0 | 0 |  |  |  |
| Coffee, tea | Positive health effects of reasonable consumption. | 680 | 12 | Filter coffee, prepared | 500 | 9 |
|  |  |  |  | Black tea, prepared | 100 | 2 |
|  |  |  |  | Green tea, prepared | 80 | 1 |

^a^ Raw weight, bone-free. The recommendation provided by the Norwegian Food-Based Dietary Guidelines is more commonly expressed in cooked weight (≤ 500 g/week).

^b^ Raw weight, bone-free. The recommendation provided by the Norwegian Food-Based Dietary Guidelines does not specify if this amount is raw or cooked weight. Cooked weight was assumed and converted to raw weight using a conversion factor of 1.213 (the average of the conversion factors for fatty and lean fish provided in the report “Measurements, weight and portion sizes for food products”^(5)^).

**Table S2**. Overview of the EAT-Lancet healthy reference diet (at 10 MJ)^(2)^ and foods included in the modelled diet scenario.

| **Guidelines:** | | | **Included in scenario:** | | | | |
| --- | --- | --- | --- | --- | --- | --- | --- |
|  | *g/day* | *kJ/day* | *g/day* | *kJ/day* |  | *g/day* | *kJ/day* |
| Total | 1268 | 10000 | 1221^a^ | 10000 |  | 2331 | 10000 |
| Whole grains^b^ | 222 (0-60% total energy content) | 3424 | 275  (whole grains: 222) | 3488 | Brown rice, dry | 45 | 694 |
|  |  |  |  |  | Wholegrain bread | 140 | 1361 |
|  |  |  |  |  | Wholegrain pasta, dry | 10 | 153 |
|  |  |  |  |  | Rolled oats, dry | 70 | 1089 |
|  |  |  |  |  | Wholegrain crispbread | 10 | 191 |
| Tubers or starchy vegetables | 48 (0-96) | 156 | 48 | 163 | Winter potatoes, raw | 48 | 163 |
| Vegetables |  |  |  |  |  |  |  |
| All vegetables | 287 (191-574) | 314 | 287 | 329 |  |  |  |
| Dark green vegetables | 96 | 92 |  |  | Broccoli, raw | 96 | 115 |
| Red and orange vegetables | 96 | 120 |  |  | Tomato, raw | 16 | 12 |
|  |  |  |  |  | Carrot, raw | 80 | 120 |
| Other vegetables | 96 | 100 |  |  | Cucumber, raw | 30 | 13 |
|  |  |  |  |  | Iceberg lettuce/ruccola, raw | 25 | 15 |
|  |  |  |  |  | Onion, raw | 40 | 54 |
| Fruits and berries | 191 (96-287) | 504 | 191 | 471 | Orange, raw | 46 | 72 |
|  |  |  |  |  | Apple, raw | 100 | 199 |
|  |  |  |  |  | Banana, raw | 40 | 141 |
|  |  |  |  |  | Raisins | 5 | 59 |
| Dry beans, lentils, peas | 48 (0-191) | 688 | 48 | 676 | Chickpeas, dry | 38 | 546 |
|  |  |  |  |  | Green/brown lentils, dry | 10 | 130 |
| Soy foods | 24 (0-48) | 448 | 24 | 384 | Soy beans, dry | 24 | 384 |
| Peanuts | 24 (0-72) | 568 | 24 | 619 | Peanuts, raw | 24 | 619 |
| Tree nuts | 24 | 596 | 24 | 593 | Cashews, raw | 14 | 344 |
|  |  |  |  |  | Almonds, skin-on, raw | 10 | 249 |
| Beef and lamb | 7 (0-13) | 60 | 7 | 32 | Beef, 2% fat, raw | 7 | 32 |
| Pork | 7 (0-13) | 60 | 7 | 38 | Ham roast, raw | 7 | 38 |
| Poultry | 28 (0-55) | 248 | 28 | 131 | Chicken filet, skinless, raw | 28 | 131 |
| Eggs | 12 (0-24) | 76 | 12 | 74 | Egg, raw | 12 | 74 |
| Fish and shellfish | 27 (0-191) | 160 | 27 | 153 | Salmon, farmed, raw | 10 | 94 |
|  |  |  |  |  | Cod, wild, raw | 17 | 59 |
| Whole milk or derivative equivalents | 239 (0-478) | 612 | 160^c^ | 665 | Milk, 4% | 100 | 267 |
|  |  |  |  |  | Yoghurt, plain, 3% fat | 40 | 116 |
|  |  |  |  |  | White cheese, 26% fat | 20 | 282 |
| Lard or tallow | 5 (0-5) | 1441 | 0 | 0 |  |  |  |
| Vegetable oils and margarine |  |  |  |  |  |  |  |
| Palm oil | 7 (0-7) | 240 | 0 | 0 |  |  |  |
| Unsaturated oil^d^ | 38 (19-76) | 1416 | 44 | 1612 | Rapeseed oil | 11 | 403 |
|  |  |  |  |  | Sunflower oil | 11 | 399 |
|  |  |  |  |  | Olive oil | 11 | 403 |
|  |  |  |  |  | Soy oil | 11 | 407 |
| All sweeteners | 30 (0-30) | 480 | 29^e^ | 650 | White sugar | 10 | 170 |
|  |  |  |  |  | Gummy candy mix, no chocolate | 15 | 243 |
|  |  |  |  |  | Coca cola, sugar-sweetened | 100 | 180 |
| Water | - | - | 1006 | 0 | Tap water | 1000 | 0 |

^a^ Minus liquid weight;

^b^ Whole grains in dry weight. In modelled scenario, whole grains are provided as a mixture of dry and finished weight;

^c a^Converted from whole milk equivalents using milk equivalent factors 1.0 (yoghurt) and 5.0 (cheese). From Wood A, Gordon LJ, Röös E et al (2019) Erratum: Nordic food systems for improved health and sustainability - baseline assessment to inform transformation. Stockholm Resilience Centre. https://www.stockholmresilience.org/download/18.8620dc61698d96b1901719c/1561013818461/Erratum_Nordic%20report_14-6-19.pdf. Accessed 29 March 2023.

^d^ Unsaturated oil recommended to be 20% each of rapeseed, sunflower, soy, peanut and olive oil. Peanut oil excluded due to lack of data availability;*

^e^ Added sugar in grams.

**Table S3.** Correlation matrix of spearman correlation coefficients for greenhouse gas emission and terrestrial acidification, freshwater eutrophication, marine eutrophication, land use and water use for current dietary intake in a sample of 1787 Norwegian adults aged 18-70 years^(3)^.

|  | **Global warming pot.** | **Freshwater eutr.** | **Marine eutr.** | **Terrestrial acid.** | **Water use** | **Land use** |
| --- | --- | --- | --- | --- | --- | --- |
| **Global warming pot. (**kg CO_2_-eq) | 1.0000 |  |  |  |  |  |
| **Freshwater eutr.**  **(**g P-eq) | 0.8961* | 1.0000 |  |  |  |  |
| **Marine eutr.**  **(**g N-eq) | 0.7581* | 0.6208* | 1.0000 |  |  |  |
| **Terrestrial acid.**  **(**g SO_2_-eq) | 0.9341* | 0.9056* | 0.5712* | 1.0000 |  |  |
| **Water use**  **(**m^3^) | 0.5754* | 0.5357* | 0.5508* | 0.5351* | 1.0000 |  |
| **Land use**  **(**m^2^a) | 0.8936* | 0.6972* | 0.7859* | 0.7851* | 0.4547* | 1.0000 |

Abbreviations: Global warming pot., global warming potential; CO_2_-eq, carbon dioxide equivalents; Freshwater eutr., freshwater eutrophication; P-eq, phosphorous equivalents; Marine eutr., marine eutrophication; N-eq, nitrogen equivalents; Terrestrial acid., terrestrial acidification; SO_2_-eq, sulfur dioxide equivalents; m3, cubic meters; m^2^a, square meters of land per year.

* P<0.0001.

**Table S4.** Per capita environmental impact of dietary consumption in a sample of 1787 Norwegian adults aged 18-70 years^(3)^, and in modelled diets representing the Norwegian Food-Based Dietary Guidelines and the EAT-Lancet healthy reference diet, compared to environmental boundaries downscaled from the EAT-Lancet targets using an equal per capita approach^(4)^.

| **Environmental indicator^a^** | **Greenhouse gas emissions** (tCO_2_-eq/yr) | **Cropland use** (hectares) | **Bluewater use** (m^3^/yr) |
| --- | --- | --- | --- |
| **Per capita boundaries^b^** | **0.69 (2015)**  **0.54 (2050)** | **0.18 (2015)**  **0.14 (2050)** | **340 (2015)**  **270 (2050)** |
| Current diet in Norway^c^ | 1.7 (1.2 – 2.0) | 0.19 (0.12 – 0.23) | 182 (110 – 256) |
| Norwegian Food-Based Dietary Guidelines^d^ | 1.58 | 0.18 | 142 |
| EAT-Lancet healthy reference diet^d^ | 0.93 | 0.14 | 113 |

^a^ The EAT-Lancet Commission Report^(2)^ also includes environmental boundaries for nitrogen and phosphorous application; these environmental indicators have been excluded from the comparison as the units of measurement given for the environmental boundaries are not directly comparable with those used in the present study.

^b^ The top number provides a boundary based on the 2015 population, and the bottom number provides a boundary based on the 2050 population.

^c^ Environmental impact per 9.3 MJ (mean energy consumption).

^d^ Environmental impact per 10 MJ.

**Table S5.** Relationship between energy-adjusted daily dietary environmental impact and gender/level of educational attainment in a sample of 1787 Norwegian adults aged 18-70 years^(3)^, results from multivariate linear regression analysis.

|  | **Women^a^** | | **Low educational attainment^b^** | | **Moderate educational attainment^b^** | |
| --- | --- | --- | --- | --- | --- | --- |
|  | **b value^c^** | **P value** | **b value^c^** | **P value** | **b value^c^** | **P value** |
| **Global warming pot.**  kg CO_2_-eq | -0.049 | 0.001* | -0.027 | 0.191 | -0.012 | 0.469 |
| **Freshwater eutr.**  g P-eq | -0.053 | 0.000* | -0.052 | 0.009* | -0.035 | 0.029* |
| **Marine eutr.**  g N-eq | -0.022 | 0.058 | -0.027 | 0.097 | -0.034 | 0.008* |
| **Terrestrial acid.**  g SO_2_-eq | -0.042 | 0.029* | -0.016 | 0.547 | 0.000 | 0.993 |
| **Water use**  m^3^ | 0.034 | 0.061 | -0.082 | 0.002* | -0.030 | 0.148 |
| **Land use**  m^2^a | -0.068 | 0.001* | 0.024 | 0.404 | 0.029 | 0.204 |

Abbreviations: Global warming pot., global warming potential; CO_2_-eq, carbon dioxide equivalents; Freshwater eutr., freshwater eutrophication; P-eq, phosphorous equivalents; Marine eutr., marine eutrophication; N-eq, nitrogen equivalents; Terrestrial acid., terrestrial acidification; SO_2_-eq, sulfur dioxide equivalents; m3, cubic meters; m^2^a, square meters of land per year; IC, impact category.

Due to the strong dependence between energy consumption and gender, the regression analysis was performed in two steps. The first regression took into account the sole effect of energy intake on IC values - with energy consumption as the independent variable and IC values as the dependent variables. Right-skewed IC variables were log transformed and the variable for energy consumption was normalized to a scale of 0-1. In the second regression step, the additional effect of gender on residual IC values was quantified, after correction for energy intake. New variables were created from the residuals of these regressions; these variables represent the component of the IC values that cannot be explained by energy intake. These variables were then entered into regression models as dependent variables with gender and educational attainment as the independent variables. This method allowed for description of the effect of gender on IC values beyond and independently of energy intake. Analyses were performed using the following models: 1: ICi = b1·energy + b; 2: Resi = b2 ·gender + b3·educational attainment + b.

^a^ Reference group: men.

^b^ Educational attainment: Low (primary school or lower), moderate (high school, trade school), high (university and above). Reference group: high educational attainment.

^c^ Unstandardized coefficient.

^*^ P<0.05.

**Figure S1.**  Relative contributions (% of total/day) of food categories to daily energy consumption in a sample of Norwegian adults aged 18-70 years^(3)^ and in modelled scenario diets representing the Norwegian Food-Based Dietary Guidelines (FBDG) and the EAT-Lancet healthy reference diet (EAT-Lancet). System boundaries are farm-to-retail.


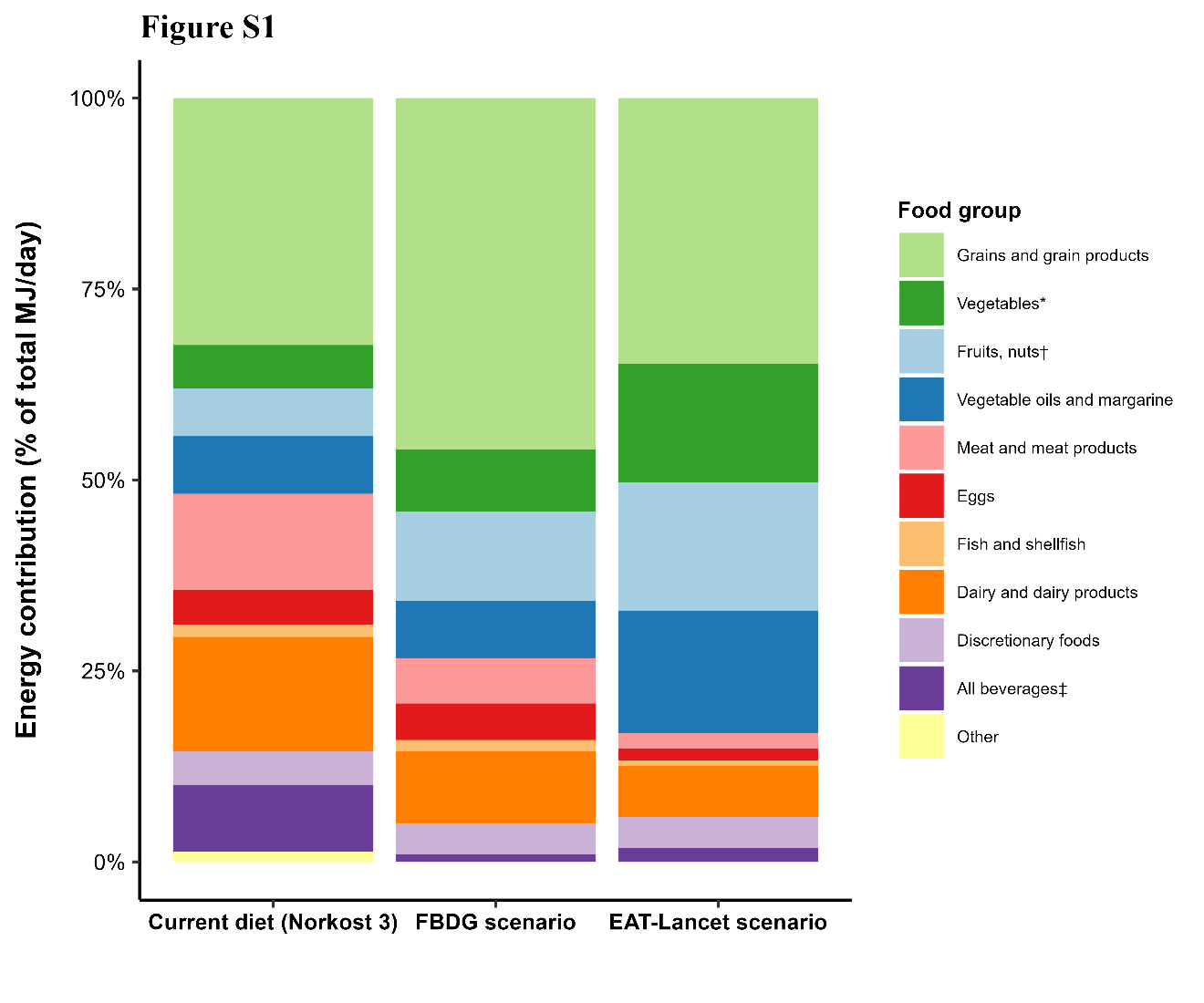


*All vegetables incl. potatoes, legumes (excl. peanuts). †Fruits, nuts, berries, seeds. Incl. peanuts. ‡All beverages incl. juice, coffee, tea, alcohol, soft drinks.

**References**

1. Norwegian Directorate of Health. Recommendations on diet, nutrition, and physical activity (In Norwegian). 2014.

2. Willett W, Rockström J, Loken B*, et al.* Food in the Anthropocene: the EAT-Lancet Commission on healthy diets from sustainable food systems. Lancet. 2019;393(10170):447-92.

3. Totland TH, Melnæs BK, Lundberg-Hallén N*, et al.* Norkost 3: A nationwide dietary survey among men and women in Norway aged 18-70, 2010-2011. (In Norwegian). 2012.

4. Wood A, Gordon, L. J., Röös, E., Karlsson, J., Häyhä, T., Bignet, V., ... & Bruckner, M. Nordic food systems for improved health and sustainability: Baseline assessment to inform transformation. . Stockholm Resilience Centre; 2019.

5. Østerholt Dalane J, Martinsen Bergvatn TA, Kielland E*, et al.* Measurements, weight, and portion sizes for foodstuffs (In Norwegian). In: Norwegian Food Safety Authority, University of Oslo, The Norwegian Directorate of Health, editors.: Wittusen & Jensen; 2015. p. 20.
